# Development and Evaluation of the Caring Ahead: Preparing for End of Life in a Dementia Questionnaire

**DOI:** 10.1093/geroni/igaa057.055

**Published:** 2020-12-16

**Authors:** Pamela Durepos, Sharon Kaasalainen, Jenny Ploeg, Tamara Sussman, Noori Akhtar-Danesh

**Affiliations:** 1 McMaster University, Hamilton, Ontario, Canada; 2 McGill University, Montreal, Ontario, Canada

## Abstract

A palliative approach is recommended in long-term care to support persons with dementia and help families prepare for end-of-life. Despite this, 50% of family caregivers of persons with dementia report feeling unprepared for death. A questionnaire is needed to assess caregiver death preparedness as an outcome measure for strategies within palliative care. A mixed methods design with qualitative and quantitative phases was used to develop and evaluate the ‘Caring Ahead: Preparing for End-of-Life in Dementia’ questionnaire. The questionnaire has 30 items organized into Medical, Relationship/Personal, Spiritual and Practical subscales with a 7-pt Likert response scale. To date, the questionnaire has been tested with 117 participants who are 61 years old on average, female (86%), adult children (77%) caring for a person with dementia in long-term care. The mean item score is 5.61 (SD 0.71). Participants report limitations in preparedness related to: 1) communication with healthcare providers about traditions and preferences for end-of-life care; 2) knowledge of the dying process and; 3) life purpose after death. A test-retest with 32 participants demonstrates a high degree of reliability; Intraclass Correlation Coefficient 0.91 (CI95%: 0.31-0.97). A moderate positive correlation between participant total scores and a single global preparedness item suggests concurrent validity, r=.66 (CI95%: 0.51-0.80). These findings will be used to refine the questionnaire and contribute a valuable measurement tool for clinicians, researchers and policy-makers working in palliative care.

